# Vitamin D Postpartum Concentrations: Relationship with Nutritional Condition and Morbidities during Pregnancy

**DOI:** 10.1155/2018/1070528

**Published:** 2018-09-02

**Authors:** Maria Eliana Pierre Martins, Carmem Ulisses Peixoto Esmeraldo, João Paulo Duarte Sabiá, João Hallisson Lemos Carvalho, Fabíola Isabel Suano-Souza, Roseli Oselka Saccardo Sarni

**Affiliations:** ^1^Associate Professor of Pediatrics at Universidade Federal do Cariri-UFCA, Brazil; ^2^Master in Science of Faculdade de Medicina do ABC, Brazil; ^3^Chemist at Universidade Potiguar, Brazil; ^4^Associate Professor at Faculdade de Medicina do ABC and Universidade Federal de São Paulo, Brazil; ^5^Full Professor at Faculdade de Medicina do ABC, Brazil

## Abstract

**Objective:**

To evaluate postpartum vitamin D deficiency/insufficiency and to relate it to pregestational BMI, gestational weight gain, and sociodemographic variables.

**Methods:**

This is a cross-sectional study with 225 full-term pregnant women. Data collected are as follows: maternal health, socioeconomic status, pregestational body mass index (BMI), and gestational weight gain. Laboratory evaluation included vitamin D [25(OH)D], calcium, phosphorus, magnesium, and alkaline phosphatase.

**Results:**

The mean age of women was 25.6±6.6 years. Dark skin color, low education, and work in the urban region were predominant. Regular sun exposure, photoprotection, and vitamin D supplementation were reported by 144 (64.0%), 44 (19.6%), and 5 (2.2%) women, respectively. The mean plasma concentrations of 25(OH)D were 26.0±6.8 ng/mL. Levels compatible with deficiency (<20 ng/mL) and insufficiency (20-30 ng/mL) were observed in 43 (19.1%) and 124 (55.1%) women, respectively. The increase of 1 ng/mL in 25(OH)D concentrations was associated with an increase of 0.16 mg/dL (95%CI 0.19 to 2.02; p=0.018) for calcium. There were no associations with 25(OH)D concentrations with pregestational BMI and with gestational weight gain.

**Conclusions:**

The high frequency of postpartum vitamin D deficiency/insufficiency in women with a full-term pregnancy in a region with a large and permanent sun exposure reminds us of the need for intervention policies aimed at preventing vitamin D deficiency during pregnancy.

## 1. Introduction

Pregnancy is a dynamic period with anatomical and physiological changes for women and their developing fetus. Vitamin D deficiency is a severe public health problem affecting around 1 billion people worldwide [1, 2]. Pregnant women are identified as risk groups for deficiency even in regions with adequate sun exposure [3–5].

The first weeks of gestation experience a twofold-threefold increase in the concentrations of 1.25(OH)_2_D with immunomodulatory effects [6, 7]. As pregnancy progresses from the end of the first trimester to puerperium, an increased concentration of 25(OH)D and 1.25(OH)_2_D occurs to ensure adequate calcium supply to the fetal skeleton [7, 8]. The mechanisms involved in this increase have not yet been fully elucidated. However, the participation of estrogen [5] is postulated.

Given the short biological half-life of 1.25(OH)_2_D, the nutritional condition for vitamin D is usually determined by serum concentrations of 25(OH)D, the primary circulating form of this vitamin in the blood [8, 9].

Vitamin D insufficiency during pregnancy contributes to adverse outcomes, such as gestational diabetes, preeclampsia, preterm birth, and fetal intrauterine growth restriction, among others [3, 4, 10]. Deficiency and insufficiency are defined by concentrations of 25(OH)D below 20.0 ng/mL (50.0 nmol/L) and 30.0 ng/mL (75.0 nmol/L), respectively [11]. Studies suggest that concentration above 40 ng/mL during pregnancy could be effective in reducing the risk of prematurity [12].

A recent systematic review described the overall mean prevalence levels of vitamin D deficiency of 54% and 75% in pregnant women and newborns, respectively [13]. In postpartum, the prevalence of deficiency in women are also high at 63% average [13, 14].

While evidence points to the high prevalence of deficiency, there is still no consensus on the evaluation of the nutritional condition related to vitamin D and the need for routine supplementation for pregnant women [15, 16]. In Brazil there is no recommendation for universal supplementation during pregnancy.

Ethnicity, latitude, season, sunscreen, and body mass index influence vitamin D concentrations [2].The maternal nutritional status, as measured by prior body mass index and gestational weight gain, may influence serum concentrations of vitamin D. Gestational obesity is associated with a reduced placental transfer of this vitamin to the fetus, bioavailability, and mother and child serum levels [17].

Considering the lack of studies that evaluated vitamin D concentrations of women in early puerperium in our country, especially in regions with high sun exposure and low economic status, we carried out this study to evaluate maternal postpartum vitamin D concentrations and to relate it to pregestational BMI, gestational weight gain, and sociodemographic characteristics.

## 2. Materials and Methods

A cross-sectional study was carried out between October and December 2016, in a reference maternity ward in the city of Crato, rural area of the State of Ceará (latitude 07° 14′ 03′′ S and longitude 39° 24′ 34′′ W). The city's climate is mostly tropical throughout the year, with temperatures ranging from 24°C to 27°C, with a peak of 33°C and a low of 18°C.

This is a sample of convenience that included consecutively 225 postpartum mothers. Pregnant women with endocrinological (other than diabetes), rheumatologic, and renal diseases were excluded from the study, as were those who used medications that interfered with the metabolism of vitamin D, such as diuretics, antihypertensives, and immunosuppressants (corticosteroids) and those who had deliveries in less than 37 weeks of gestational age.

The study observed the ethical precepts of the Declaration of Helsinki and Resolution 510/2016 of the Brazilian National Health Council, and participating women signed the Informed Consent Form (ICF). The Human Research Ethics Committee of the Faculty of Medicine of the ABC, Santo André, approved the study under opinion n° 1.813.560.

### 2.1. Data Collected

#### 2.1.1. Maternal Socioeconomic Status and Gestational Data

Standardized survey containing information on life habits, socioeconomic status, use of medications, personal and obstetrical background, and previous diseases developed during pregnancy was applied to women. Regarding the vitamin D related nutritional status, we investigated the use of vitamin/mineral supplements, skin color, frequency of sun exposure, and photoprotection. The prenatal examination card was also checked to collect information on the development of gestation, the date of the last menstrual period, anthropometric measurements, and laboratory tests.

The body mass index (BMI, kg/m^2^) was calculated based on the weight and height measures contained in the card and was classified according to the gestational age as low, adequate, overweight, and obesity. Gestational weight gain was also noted, and the cutoff proposed by the Institute of Medicine were used for classification based on prepregnancy BMI (underweight: 13 a 18 kg, normal weight: 11 a 16 kg, overweight: 7 a 11 kg, and obese: 5 a 9 kg) [18].

### 2.2. Laboratory Tests

Blood samples were collected from women in a rooming-in setting, within 24 hours of delivery. We collected 1.5 mL of blood via peripheral venipuncture, which was packed in vials with photoprotection and transferred under refrigeration to the clinical laboratory for centrifugation and analysis. Concentrations of 25(OH)D were determined by electrochemiluminescence with UniCel DXI 800 Immunoassay System, Beckman Coulter. Calcium, phosphorus, magnesium, and alkaline phosphatase concentrations were evaluated by spectrophotometry using the Vitros 5600 Integrated System, Ortho-Clinical Diagnostics. Plasma concentrations of 25(OH)D below 20 ng/mL were defined as deficiency, between 20 ng/mL and 30 ng/mL as insufficiency and above 30 ng/mL as sufficiency.

All calculations followed good clinical practice. The average of 19.9 ng/mL (SD: 0.948 ng/mL and an intracontrol CV of 4.8%) was the referred reproducible one for measuring 25(OH)D. For the same parameter, the intermediate precision was 19.9 ng/mL (SD: 1.23 ng/mL and an intracontrol CV of 6.2%).

### 2.3. Statistical Analysis

A spreadsheet was built in Excel® Office containing information on identification, general characteristics, questionnaire data on pregnant women, anthropometric data, and laboratory test results. The spreadsheet was revised, consolidated, and imported in the SPSS 24.0 statistical package (IBM®). Categorical variables were shown as absolute and percentage numbers and compared by the Chi-square test. Continuous variables were tested for their normality, shown as mean (standard error) and compared using Student's* t*-test. The linear regression Enter method was used for multivariate analysis. Statistical significance is claimed for* p* < 0.05.

## 3. Results

The mean age of women included in the study was 25.6±6.6 years (range: 15.3 to 44.3 years). A predominance of dark skin (178, 79.1%), low education (less than four years, 197, 87.6%), and working in the urban area (127, 56.4%) was observed. One hundred and eight (48.0%) women were primiparous (Table 1).

Regarding prenatal care, all women had at least one visit, and 189 (84%) attended more than six visits. Regular sun exposure, use of photoprotection, and vitamin D supplementation were reported by 144 (64.0%), 44 (19.6%), and five (2.2%) of the women evaluated, respectively (Table 1). The vitamin D supplements contained colecalciferol concentration of 250 to 400 UI.

The main complication during gestation was the urinary tract infection (72 cases, 32%), followed with a smaller proportion of pregnancy-specific hypertensive disease (20 cases, 8.9%), gestational diabetes (2 cases, 0.9%), and bleeding (17 cases, 7.6%) (Table 1). The mean gestational age was 39.1±1.1 weeks, with mostly surgical deliveries (164, 72.9%).

Regarding nutritional status, pregestational BMI was compatible with malnutrition, overweight, and obesity in 19 (8.5%), 49 (21.9%), and 28 (12.0%) women, respectively. Gestational weight gain was low, adequate, and higher than recommended in 83 (36.9%), 70 (31.1%), and 71 (31.6%) women, respectively (Table 1).

The combined analysis of gestational weight gain according to the previous nutritional condition showed that only 11 (13.3%), 42 (50.6%), 20 (24.1%), and 10 (12.0%) women with malnutrition, normal weight, overweight, and obesity gained weight as recommended (Table 1). Gestational weight gain was more appropriate for women with normal pregestational BMI (42, 50.6%) (p <0.001).

The mean maternal postpartum 25(OH)D plasma concentrations were 26.0±6.8 ng/mL (range: 8.4 to 52.5 ng/mL). Levels compatible with deficiency (<20 ng/mL) and insufficiency (20 to 30 ng/mL) were observed in 43 (19.1%) and 124 (55.1%) women, respectively. Thus, only 58 (25.8%) had sufficient concentrations of 25(OH)D (>30 ng/mL) (Table 1).

**Figure 1:**
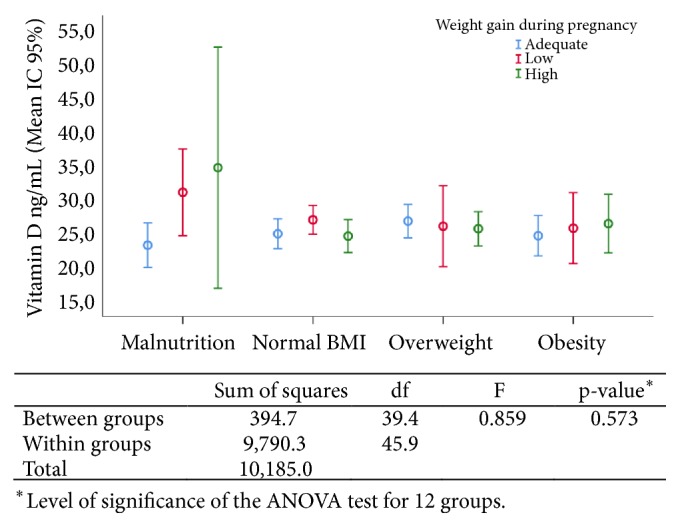
Mean of 25(OH)D concentrations concerning the maternal nutritional status before and gestational weight gain.

There was no association of 25(OH)D concentrations with pregestational BMI nor with gestational weight gain, when these indicators were evaluated separately and in combination, as shown in Figure 1.

The maternal postpartum 25(OH)D concentrations correlated directly those of calcium (r=0.173, p=0.009) and alkaline phosphatase (r=0.149; p=0.025). Multivariate analysis showed that each increase of 1 ng/mL in 25(OH)D concentrations was associated with an increase of 0.16 mg/dL (95% CI 0.19 to 2.02, p=0.018) for calcium.

Table 2 compared the maternal postpartum 25(OH)D levels below and above 30 ng/mL. The 25(OH)D insufficiency (< 30 ng/mL) was not associated with any of the clinical and socioeconomic studied variables.

## 4. Discussion

In this study postpartum vitamin D insufficiency was observed in 74% of women. There was no association of insufficiency with maternal pregestational BMI and gestational weight gain. It should be noted that this study was collected during the spring in the region of Crato, where ultraviolet radiation is high throughout the year.

Seasonal variation of vitamin D predominates in regions with higher latitude and better defined seasons, such as, for example, in European countries such as Ireland (52° N) or Poland (52° N), with lower concentrations of vitamin D during winter [19, 20].

The high prevalence of deficiency/insufficiency, particularly in low-income countries such as those observed by us, has implications for mother and child health, with data supported by two meta-analyses [21, 22]. Showing the global situation of vitamin D deficiency in pregnant women and newborns, Saraf et al. [13] emphasized the relevance of the subject as a priority agenda for public health intervention, describing deficiency in 54% of pregnant women and 75% of newborns, respectively.

In our study, despite low frequency of vitamin D supplementation during pregnancy, postpartum deficiency was lower than other studies with similar population. The fact of our study has been performed in a place with high insolation rate, during the spring and summer seasons, and women often expose themselves to the sun could be explain this lower prevalence of deficiency found [20, 22, 23]. The collection of 25(OH)D within the first 24 hours of delivery may also justify this finding; it is known that pregnant women have about twice as much more circulating vitamin D metabolites compared to nonpregnant women [6, 19].

A cohort study with 229 Brazilian pregnant women described the evolution of 25(OH)D and 1.25(OH)_2_D concentrations during pregnancy. The prevalence of insufficiency was 70.4%, similar to that observed by us. Pregnant women who started pregnancy in winter, autumn and spring showed progressive elevation in the concentrations of both metabolites, something not observed for those who became pregnant in the summer. The increase was also higher in those who started pregnancy with deficiency/insufficiency compared to those with sufficiency. Like our findings, there was no association with pregestational BMI [8].

There are few studies that evaluated vitamin D concentrations in the immediate puerperium. A Jordanian study (n=171) described deficiency and insufficiency in 76% and 24% of puerperae, respectively. Contrary to the observed by us, the authors described association between low 25(OH)D concentrations with multiparity and breastfeeding [5].

Strategies to raise postpartum vitamin D concentrations include supplementation, advice for sun exposure (15-20% of body surface area), and intake of source foods. There is still no consensus on the dose that should be used for the supplementation of women in gestation and puerperal period [22, 23]. In our study, maternal supplementation was not associated with better postpartum vitamin D concentrations. We speculate that the lack of association between vitamin D insufficiency and maternal vitamin D intake is due to the low number of women with vitamin D supplementation.

The identification of postpartum deficiency/insufficiency vitamin D may represent a window of opportunity for vitamin D supplementation aiming at raising vitamin D concentrations in women [24, 25].

We observed that postpartum 25(OH)D concentrations were directly related to calcium and alkaline phosphatase concentrations, similar to that observed by Abbasian et al. [26]. The lack of association between vitamin D deficiency/insufficiency, maternal nutritional status, sociodemographic and clinical variables related to vitamin D suggests the existence of other associated factors not considered in this study.

This study had some limitations. First, inclusion of pregnant women limited to spring and summer seasons, lack of a control group of nonpregnant women, single vitamin D collection during postpartum, low frequency of intake vitamin D supplements, and not including data on dietary intake and physical activity.

The high frequency of postpartum vitamin D deficiency/insufficiency in a region with extensive and permanent sun exposure reminds us of the need to discuss public intervention policies aimed at reducing the prevalence of vitamin D deficiency during the pregnancy.

## 5. Conclusion

This study identified a high frequency of postpartum 25(OH)D insufficiency in healthy not supplemented women, living in high solar incidence area. These low postpartum vitamin D concentrations were not associated with the socioeconomic factors studied and with the nutritional condition of pregnant women. Further studies are required to identify the risk factors for this high proportion of insufficiency and the possible adverse outcomes for pregnant women.

## Figures and Tables

**Table 1:** Characterization of women included in the study (n = 225).

Variable	Unit		%
General characteristics			

Mother's age	Years	25.6 ± 6.6	
Father's age	Years	28.9 ± 7.9	
Skin type	White skin	47	20.9
	Dark skin	178	79.1
Mother's employment	Housewife	55	24.4%
	Urban	127	56.4%
	Rural	43	19.1%
Mother's schooling	< 4 full years	197	87.6%
	4 to 8 full years	13	5.8%
	> 8 years	15	6.7%
Per capita income	American dollars	44,00 ± 32,00	
People living in the household	Number	4.6 ± 2.2	

Gestational conditions			

Number of pregnancies	Number	2.1 ± 1.6	
Primiparous	Yes	108	48.0%
Prenatal care visits	Number	7.8 ± 2.1	
Pregestational BMI	< 18,5 kg/m^2^	19	8.5%
	18.5 to 25 kg/m^2^	128	57.1%
	25 to 30 kg/m^2^	49	21.9%
	> 30 kg/m^2^	28	12.5%
Gestational weight gain	Adequate	83	36.9%
	Low	70	31.1%
	High	71	31.6%
Tobacco use	Yes	9	4.0%
Alcohol use	Yes	11	4.9%
Vitamin D supplement	Yes	5	2.2%
Folic acid	Yes	198	88.0%
Iron	Yes	204	90.7%
Regular sun protection	Yes	44	19.6%
Regular sun exposure	Yes	144	64.0%
	Hours/day	1.9 ± 1.0	
Complications	Urinary infection	72	32%
	GSHD	20	8.9%
	GDM	2	0.9%
	Bleeding	17	7.6%
Delivery type	Vaginal	61	27.1%
	Surgical	164	72.9%
Gestational age	Weeks	39.1 ± 1.1	

Laboratory tests			

Vitamin D 25(OH)D	ng/mL	26.0 ± 6.8	
	< 20 ng/mL	43	19.1%
	20 a 30 ng/mL	124	55.1%
	> 30 ng/dL	58	25.8%
Calcium	mg/dL	8.4 ± 0.9	
Phosphorus	mg/dL	4.5 ± 0.8	
Magnesium	mg/dL	1.8 ± 0.5	
Alkaline phosphatase	U/L	152.0 ± 43.5	

BMI: body mass index, GSHD: gestational-specific hypertensive disease, GDM: gestational diabetes mellitus.

**Table 2:** Comparison of conditions during gestation with vitamin D concentrations in women evaluated.

Variable		25OHD3 < 30 ng/mL (n=167)	25OHD3 ≥ 30 ng/mL (n=58)	p-value
Mother's age	years	25.9 ± 6.5	24.5 ± 6.6	0.165^1^
Ethnicity	Brown	132 (79.0%)	46 (79.3%)	0.564^2^
Employment type	Urban	46 (27.5%)	9 (15.5%)	0.180
Schooling	< 4 years	146 (87.4%)	51 (87.9%)	0.315
Gestations	Number	2.0 ± 1.6	2.2 ± 1.8	0.362
Primiparous	Yes	82 (49.1%)	26 (44.8%)	0.648
Pregestational BMI	Obesity	22 (13.2%)	6 (10.5%)	0.817
Gestational weight gain	High	54 (32.3%)	17 (29.8%)	0.869
Tobacco use	Yes	6 (3.6%)	3 (5.3%)	0.579
Alcohol use	Yes	10 (6.0%)	1 (1.8%)	0.202
Vitamin D supplement	Yes	14 (8.4%)	4 (7.0%)	0.498
Folic acid	Yes	147 (88.0%)	51 (89.5%)	0.768
Iron	Yes	151 (90.4%)	53 (93.0%)	0.558
Sun exposure	Yes	107 (64.1%)	37 (64.9%)	0.521
Sun protection	Yes	33 (19.8%)	11 (19.3%)	0.554
Urinary infection	Yes	52 (31.1%)	20 (35.1%)	0.623
GSHD	Yes	15 (9.0%)	5 (8.8%)	0.601
GDM	Yes	1 (0.6%)	1 (1.8%)	0.445
Gestational age	Weeks	39.1 ± 1.1	39.2 ± 1.0	0.378^1^

^1^Student *t*'s significance level. ^2^Chi-square test significance level.

BMI: body mass index, GSHD: gestational-specific hypertensive disease, GDM: gestational diabetes mellitus.

## Data Availability

The data used to support the findings of this study are included within the article.
